# The effectiveness of conization treatment for post-menopausal women with high-grade cervical intraepithelial neoplasia

**DOI:** 10.3892/etm.2012.748

**Published:** 2012-10-15

**Authors:** XIAODONG CHENG, YAN FENG, XINYU WANG, XIAOYUN WAN, XING XIE, WEIGUO LU

**Affiliations:** Department of Gynecologic Oncology, Women’s Hospital, School of Medicine, Zhejiang University, Hangzhou, Zhejiang 310006, P.R. China

**Keywords:** cervical intraepithelial neoplasia, conization, colposcopy, post-menopause

## Abstract

The aim of this study was to evaluate the effectiveness of conization in the diagnosis and treatment of high-grade cervical intraepithelial neoplasia (CIN) in post-menopausal women. A total of 101 post-menopausal patients who were diagnosed with high-grade lesion CIN by biopsy and in whom conization was used as the primary treatment were examined and 202 pre-menopausal patients were studied as the controls. Clinical and pathological data including symptoms, cytological examination and HPV DNA test results before and after conization treatment were analyzed. Both the cytological abnormalities (57.9 vs. 58.5%, P=0.260) and the positive rate of the HPV DNA test (89.5 vs. 86.4%, P=0.812) did not show a significant difference between the post- and pre-menopausal group. The rate of satisfactory colposcopy was significantly lower in post-menopausal patients compared with pre-menosausal patients (23.2 vs. 68.9%, P<0.001). Post-menopausal patients presented a significantly lower diagnostic consistency between colpscopy-directed biopsy and conization (46.4 vs. 68.9%, P=0.004), and a significantly higher positive margin rate of conization (20.8 vs. 10.9%, P=0.020). A total of 10 of the 101 post-menopausal and 2 of the 202 pre-menopausal women were diagnosed with invasive cancer by conization and underwent further treatment. In conclusion, these data suggest that conization, as a conservative primary treatment, is not suitable for post-menopausal women with high-grade lesion CIN due to the lower rate of satisfactory colposcopy, lower consistency of diagnosis between colposcopy-directed biopsy and conization, and a higher positive margin of conization.

## Introduction

Cervical intraepithelial neoplasia (CIN) is the potentially premalignant transformation of squamous cells on the surface of the cervix. CIN is usually curable with most CIN cases remaining stable or being eliminated by the immune system. However, a small portion of CIN cases, if left untreated, may develop into cervical cancer (1). The infection of the cervix with human papillomavirus (HPV), particularly high-risk HPV (HR-HPV) type 16 or type 18, is the major cause of CIN (2). According to the disease staging standard, CIN can be divided into 2 categories: low-grade lesion (CIN1) and high-grade lesion (CIN2 and CIN3) (3). The immediate treatment of CIN2 and CIN3 is usually necessary as the spontaneous regression rates at these stages are low (32–43%) and, if such a disease is left untreated, the risk of progression to invasive cancer is substantially increased by 5–22% (4,5).

The American Society for Colposcopy and Cervical Pathology (ASCCP) issued an updated consensus for the management and treatment of cervical cytological abnormalities in 2006. These guidelines act as the professional standard for curing CIN or adenocarcinomas (3). According to the treatment recommendations for CIN2 and CIN3, for biopsy-confirmed cases with good colposcopy results, either ablative or excisional treatments may be chosen. However, for the cases with unsatisfactory colposcopy results, only excisional treatments are available. The excisional treatments include loop electrosurgical excision procedure (LEEP), cold-knife conization (CKC) and laser conization. Among those methods, LEEP and CKC are 2 procedures used most frequently (3,6). The post-menopausal female patients have quite different physiological characters from the pre-menopausal female patients. For example, post-menopausal female patients usually have atrophy of the cervix, the transformation zone is not easily observed by colposcopy and there are increased conizaion treatment difficulties. Therefore, the effectiveness of the treatment of high-grade CINs (CIN2 and CIN3) in post-menopausal women needs to be investigated.

In this study, we analyzed the conization treatment effectiveness of patients at CIN2 or CIN3 stages. The association between clinical and pathological data from the pre-menopausal patients was also studied to evaluate conization treatment effectiveness for post-menopausal women with high-grade CINs.

## Patients and methods

### Patients

A toal of 101 menopausal patients with high-grade CIN (CIN2 and CIN3) confirmed by histological analysis were enrolled in this study, including 56 cases diagnosed by colposcopy-directed biopsy and 45 cases by multiple biopsy, at the Women’s Hospital, School of Medicine, Zhejiang University (Zhejiang, China), from January 2008 to October 2010. All patients received conization (LEEP in 44 cases and CKC in 57 cases) as the primary therapy. Pre-menopausal patients with CIN2 and CIN3 confirmed by histological analysis (119 cases diagnosed by colposcopy-directed biopsy and 83 cases by multiple biopsy) were examined as the controls (the ratio of post- to pre-menopausal cases was 1:2). In toal, 202 pre-menopausal patients were enrolled in the study. The principles of pairing were as follows: i) the same histological grade lesion diagnosed by biopsy; ii) the same period of treatment (±2 weeks); and iii) the same method of excision (LEEP or CKC). Written and informed consent was obtained from every patient and the study was approved by the ethics review board of Zhejiang University

### Pap smear examination

The clinical and pathological data, including symptoms such as abnormal bleeding, leukorrhagia, soreness of the loins, cytological data and the HPV test prior to biopsy, conization outcomes and subsequent therapy following conization, were retrospectively analyzed between the cases and controls. Samples of exfoliated cervical cells were collected with a cervical sampler during gynecological examinations prior to diagnosis. Liquid-based cervial cytology was performed (Cytyc Corp., Boxborough, MA, USA) and the Bethesda System (2001) was used for cytological diagnosis.

### HPV DNA test

Cervical specimens were collected with a cervical sampler during gynecological examinations. The HPV DNA test was performed using the Hybrid Capture II method (Qiagen Gaithersburg, Inc., Gaithersburg, MD, USA).

### Statistical analysis

Categorical variables were analyzed with Pearson’s χ^2^ test. Statistical analyses were performed using SPSS version 16 software (SPSS, Inc., Chicago, IL, USA) and P<0.05 was considered to indicate a statistically significant difference.

## Results

### Positive rate of HR-HPV in post-menopausal patients is similar to that in pre-menopausal patients

From January 2008 to October 2010, 1,810 women were diagnosed with high-grade CIN by cervical biopsy and histological analysis, and received therapy. Among them, 119 patients were post-menopausal and 101 of them received excisional conization (LEEP or CKC) as the primary treatment. The average age of the patients was 56 years (age range, 46–78 years), with the menopausal period varying from 1 to 30 years. Among the 1,810 patients, there were 4 patients who were 70 years or older. The average age of the pre-menopausal patients was 36 years (age range, 20–51 years).

In the post-menopausal patients, the main symptoms included abnormal bleeding (24.7%), leukorrhagia (8.9%) and soreness of the loins (6.9%). In pre-menopausal patients, the main symptoms were similar, including abnormal bleeding (26.2%), leukorrhagia (16.3%) and soreness of the loins (2.5%). Approximately half of the post- and pre-menopausal patients did not experience any symptoms. There were no significant differences between the 2 groups of patients (59.4 vs. 54.9%, χ^2^=0.54, P=0.461).

In total, 76 out of the 101 post-menopausal women and 171 out of the 202 pre-menomausal women received a Pap smear examination. The results from the cytological analysis demonstrated a 57.9% diagnostic consistency between the cytology and biopsy histology in the post-menopausal patients and 58.5% in the pre-menopausal patients; there was no significant difference between the 2 groups (χ^2^=1.27, P=0.260). As demonstrated in Table I, 76 out of the 101 post-menopausal women and 173 out of the 202 pre-menopausal women received a HPV DNA test. The results revealed that the positive rate of HR-HPV was 89.5% in the post-menopausal patients and 86.4% in the pre-menopausal patients. No significant difference was observed between the 2 groups (χ^2^=0.06, P=0.812).

### Rate of satisfactory colposcopy is significantly lower in the post- than in pre-menopausal patients

A total of 56 post-menopausal patients received colposcopy, out of which 6 were diagnosed without intraepithelial lesions, 14 with mild cervical intraepithelial lesions, 16 with moderate cervical intraepithelial lesions, 17 with severe cervical intraepithelial lesions and 3 as cervical cancer patients. Among the 119 pre-menopausal patients who received colposcopy, 7 were diagnosed without intraepithelial lesions, 26 with mild cervical intraepithelial lesions, 42 with moderate cervical intraepithelial lesions and 44 with severe cervical intraepithelial lesions. The rate of satisfactory colposcopy was significantly lower in the post- than in pre-menopausal patients, as demonstrated in Table II (23.2 vs. 68.9%, χ^2^=32.04, P<0.001). Taking the histological diagnosis of CIN by conization as the golden standard, the consistency of colposcopy-directed biopsy was also significantly lower in post- than in pre-menopausal patients (46.4 vs. 68.9%, χ^2^=8.14, P=0.004); the upgrading between colposcopy-directed biopsy and conization was significantly higher in post- than in pre-menopausal patients (26.8 vs. 6.8%, χ^2^=13.43, P<0.001).

### Overall positive margin rate in post-menopausal patients is significantly higher than that in pre-menopausal patients

As shown in Table III, the overall positive margin rate of conization was 20.8% in post-menopausal patients, which was significantly higher than that (10.9%) in pre-menopausal patients (χ^2^=5.42, P=0.020). The positive margin rate of CKC was 12.3% in post-menopausal patients, significantly different from the rate in pre-menopausal patients, which was only 1.8% (χ^2^=8.44, P=0.004). Furthermore, LEEP treatment had a higher positive margin rate than CKC in both the post- (31.8 vs. 12.3%) and pre-menopausal patients (22.7 vs. 1.8%); however, the positive margin rate of LEEP treatment was not significantly different between the post- and pre-menopausal patients (31.8 vs. 22.7%, χ^2^=1.27, P=0.260).

A total of 10 post-menopausal patients were diagnosed as having invasive cervical cancer following conization treatment. The cervical cancer and positive margin patients received subsequent therapy, including hysterectomy with or without adjuvant radiotherapy. Only 2 cases of invasive cervical cancer were found in the pre-menopausal patients and both received only hysterectomy. Among the 22 pre-menopausal patients with positive margin, 18 received repeated conization and 4 patients received hysterectomy.

## Discussion

In the United States, the incidence of CIN2 and CIN3 is reported to be 1.5 per 1,000 women, with the peak prevalence of lesions occurring in 25- to 35-year-old women (7). However, the real incidence of high-grade CIN remains unknown, including the incidence among post-menopausal women. In the study by Chen *et al*(8), it was reported that among 1,113 cases of CIN3, 4.3% occurred in post-menopausal women. In our study, 119 CIN2–3 cases occurred in post-menopausal women, accounting for 6.5% of the 1,810 cases. Previous data suggest that high-grade CIN is not rare in post-menopausal women. Consistently, approximately half of the high-grade CIN patients in this study, who were post-menopausal female patients, presented with no obvious symptoms. Therefore, post-menopausal women should receive regular cervical cancer screening. In addition, we found that of the 1,810 cases, only 4 women with high-grade CIN were above 70 years of age, accounting for 0.2% of all the cases. Therefore, the termination of cytological screening as guided by ASCCP, should only be considered for those women over 70 years of age.

Cytological analysis and the HR-HPV DNA test are the 2 main methods for cervical cancer screening. In this study, we found that the consistency of cytology and the HR-HPV positive rate between post- and pre-menopausal women were similar, suggesting that routine screening methods including cytology and the HPV DNA test should also be made available to post-menopausal women.

Colposcopy-directed biopsy is the golden standard for the diagnosis of cervical cancer and its precursors (9). However, the atrophy of the cervix and retraction of the squamo-columnar junction (SCJ), and reduced cellular exfoliation in post-menopausal women may lead to an unsatisfactory colposcopic examination and a decrease in the accuracy of colposcopy, and subsequently to the misdiagnosis of CIN (10). Our results revealed that the consistency of colposcopy-directed biopsy was significantly lower in post- than in pre-menopausal patients. It was also revealed that the rate of satisfactory colposcopy was significantly lower in post- than in pre-menopausal patients. A previous study revealed that pre-treatment of the cervix, such as physical manipulation of the cervix by a cotton-tipped application and use of estrogen or misoprostol prior to examination, may increase the rate of satisfactory colposcopy (11). However, more evidence supporting this hypotheis is required.

The management of high-grade CIN in post-menopausal women is not yet fully understood (12). Hysterectomy is frequently selected as the primary treatment for post-menopausal patients. An advantage of hysterectomy may be that it is easier to trace the status of the aginal cuff; however, the disadvantages may be that it is unecessary for the treatment of CIN and increased mortality with age (13). Conization is an alternative strategy for post-menopausal women. It can fulfill diagnostic and therapeutic propose at the same time; however, the positive margin is a main cause of treatment failure and limits the application of conization as a therapeutic method. Dan *et al*(14) reported that from the 23 post-menopausal patients with CIN3, 78.3% received conization treatment and the positive margin of conization was 38.9%. It has been shown by a meta-analysis that the positive margin rate of conizaion is higher in post-menopausal patients (15). Chen *et al*(8) found that the risk factors of positive margins for CIN3 patients were LEEP, carcinoma *in situ*, menopausal status and a larger lesion area. In our study, 101 out of the 119 post-menopausal women with CIN2–3 received conization using LEEP or CKC. The overall positive margin rate of conization in post-menopausal patients was as high as 20.8% and it reached 31.8% when LEEP was used. Therefore, conization as a conservative treatment is suitable for post-menopausal women with high-grade CIN, particularly when LEEP is used. However, conization can provide more exact pathological information, particularly in the evaluation of CIN grading and stromal invasion. Our results showed that the frequency of underestimation of CIN grading by colposcopy-directed biopsy was higher in post- than in pre-menopausal patients (26.8 vs. 6.7%), Therefore, these data suggest that diagnostic conization can provide guidance for selecting different types of hysterectomy for post-menopausal women following conization treatment.

In conclusion, it is recommended that post-menopausal women receive regular cervical cancer screening, and that the strategies of cervical cancer screening for post-menopausal women be the same as those for pre-menopausal women. Considering the fact of the higher frequency of unsatisfactory colpscopy and the lower consistency of histological diagnosis between colposcopy-directed biopsy and conization in post-menopausal patients, diagnostic conization may prove to be effective, and may provide guidance for selecting different types of hysterectomy for post-menopausal women.

## Figures and Tables

**Table I:** Cervical cytology results prior to biopsy in post- and pre-menopausal patients.

Cytology	Post-menopausal patients (n=76)	Pre-menopausal patients (n=171)
NILM	9	12
ASCUS	15	26
ASC-H	22	49
LSIL	11	24
HSIL	33	76
SCC	3	1
Consistency with histology by biopsy n (%)	44 (57.9%)^a^	100 (58.5%)

a LSIL and HSIL. NILM, negative for intraepithelial lesion or malignancy; ASCUS, atypical squamous cells of undetermined significance; ASC-H, atypical squamous cell, cannot exclude HSIL; LSIL, low-grade squamous intraepithelial lesions; HSIL, high-grade squamous intraepithelial lesions; SCC, squamous cell carcinoma.

**Table II:** Rate of satisfactory colpscopy and accuracy of colposcopy-directed biopsy in post- and pre-menopausal patients.

Factors	Post-menopausal (n=56) patients, n (%)	Pre-menopausal (n=119) patients, n (%)	χ^2^	P-value
Rate of satisfactory colpscopy	13 (23.2)	82 (68.9)	32.04	<0.001
Upgrading between biopsy and conization	15 (26.8)	8 (6.8)	13.43	<0.001
Consistency between biopsy and conization	26 (46.4)	82 (68.9)	8.14	0.004

**Table III:** Histology results and positive margins by conization in post- and pre-menopausal patients.

	Post-menopausal patients (n=101)			Pre-menopausal patients (n=202)		
Histology	LEEP	CKC	Total	LEEP	CKC	Total
Normal	6	9	15	8	9	17
CIN1	0	3	3	3	6	9
CIN2–3	32	41	73	75	99	174
Cancer	6	4	10	2	0	2
Positive margins (%)	14 (31.8)^a^	7 (12.3)^b^	21 (20.8)^c^	20 (22.7)	2 (1.8)	22 (10.9)

a post-menopause vs. pre-menopause: χ^2^=1.27, P=0.260;

b post-menopause vs. pre-menopause: χ^2^=8.44, P=0.004,

c post-menopause vs. pre-menopause: χ^2^=5.42, P=0.020. LEEP, loop electrosurgical excision procedure; CKC, cold-knife conization; CIN1–3, cervical intraepithelial neoplasia 1–3.
